# The Knowledge of Gynecologists and Pediatricians from Recife Public Hospitals about Hight Risk Factors for Deafness

**DOI:** 10.1590/S1808-86942010000400016

**Published:** 2015-10-19

**Authors:** Lílian Muniz, Silvio da Silva Caldas Neto, Mariana de Carvalho Leal Gouveia, Mariana Albuquerque, Andréa Aragão, Greice Mercês, Bárbara Araújo

**Affiliations:** a Doctoral degree in cognitve psychology, UFPE. Adjunct professor at the Pernambuco Federal University (Universidade Federal de Pernambuco - UFPE); b Adjunct professor, FMUSP. Adjunct professor at the UFPE; c Doctoral student in sciences - otorhinolaryngology, FMUSP. Manager of the Otorhinolaryngology Unit, Agamenon Magalhães Hospital; d Specialist in clinical audiology, Pernambuco Catholic University (Universidade Católica de Pernambuco). Speech therapist at the Otorrinos Clinic, Recife; e Speech therapist, Pernambuco Catholic University; f Speech therapist, Pernambuco Catholic University; g Specialist in clinical audiology, Pernambuco Catholic University. Speech therapist

**Keywords:** hearing, hearing loss, public health

## Abstract

Hearing is one of the main forms of connection between human being and the environment; however, hearing loss is still diagnosed very late in Brazil, which directly interferes with the child's development.

**Aim:**

The aim of this study was to check the knowledge pediatricians and gynecologists have about the risk factors for the deafness, the way they acquired such knowledge and parent education about the subject.

**Materials and Methods:**

We enrolled 119 doctors from three public hospitals of the city of Recife. An interview was applied, before and after the educational campaign on the matter. The study was descriptive, cross-sectional, case series-type. Data analysis was descriptive and inferential.

**Results:**

The results showed that only 3 of the 18 hearing loss risk factors listed had gotten answers above 50% in the initial stage of the study and 53.84% of the professionals educated the families. All the answers had increased in the second stage.

**Conclusion:**

The results emphasize the need to pay more attention to this matter; therefore, primary care is an inexpensive and efficient way to fight hearing loss.

## INTRODUCTION

Hearing is one of the main connections between human beings and their environment. This bond requires whole auditory pathways, which make it possible for certain sounds to become familiar before birth, such as the mother's heart beats and voice.^1^^,^^2^

Hearing loss is a public health issue, but it is still diagnosed late in Brazil, resulting in losses that directly affect the global development of children.^3^, ^4^, ^5^, ^6^ It may occur in the prenatal, perinatal and postnatal periods, and one of its classifications includes a division into acquired and congenital hearing losses.^7^^,^^8^

The incidence of acquired hearing loss in Brazil has increased markedly in recent years; the main reason is lack of prevention of the main infectious and contagious diseases. Consequently, pre- and perinatal hearing losses account for 65% of deafness cases.^9^^,^^4^

Neonatal hearing screening may be done universally for all live births or for children with risk factors for hearing loss. Estimates suggest that the prevalence of neonatal hearing loss is about 1 to 3 for each 1,000 neonates; this number increases to 1 to 6 for each 1,000 children admitted into neonatal intensive care units.^10^, ^11^, ^12^, ^13^^,^^6^ The fail rate in hearing screening increases at earlier gestational ages (under 30 weeks) and lower birth weights (below 1,500g). The possibility of failed hearing screening is 37 times higher in syndromic term births compared to non-syndromic term births; if a neonate has a family history of hearing loss, the chance of failed hearing screening is 7 times higher.^14^^,^^15^

About 50% of hearing losses may be suspected in the neonatal unit when hearing screening is done. Of all newborn, 7 to 12% have at least one risk factor for hearing loss; 2.5 to 5% of risk neonates have moderate to severe hearing loss^16^^,^^17^

Health care professionals should know about the factors that cause hearing loss, especially those that affect children in the prenatal, perinatal and immediately postnatal phases, so that hearing loss may be detected early. Every effort is only valid if professionals involved in neonatal care are aware and ready to set in motion diagnostic and (re)habilitation processes.^18^

Gynecologists are the first health care professionals to contact women before conception; it is therefore paramount for these professionals to know about risk factors to offer primary prevention of hearing loss.^7^ In sequence, the pediatrician first contacts the child soon after birth, followed by the family; the former is in charge of identifying children with suspected hearing loss.^7^ Otorhinolaryngologists and speech therapists - because of their training - also have a relevant role in education about hearing loss, its diagnosis and eventual interventions.^2^

These four professionals are part of a multidisciplinary team, and it is essential for them to know about the risk factors so as to help prevent hearing loss and avoid the tragic effects resulting from lack of auditory stimulation.^19^^,^^7^

The purpose of this study was to investigate the knowledge pediatricians and gynecologists have about the risk factors for hearing loss, and to underline the importance of such knowledge within the medical community. Additional aims were to check whether families were being oriented about the risk factors for hearing loss and about hearing screening for neonates, to verify how such knowledge was acquired, and to disseminate the need for multidisciplinary work.

## MATERIALS AND METHOD

This was a descriptive, cross-sectional study – a case series, with a sample of 119 medicine graduates, from both genders, with specialization or residency in gynecology and/or pediatrics. They were broken down into two groups; one was formed by 65 gynecologists and the other by 54 pediatricians. The participants were members of the clinical staff of the hospitals investigated and we included only those who participated in the two stages of the study which investigated three public reference maternal-pediatric hospitals in the city of Recife. This study was approved by the Ethics in Research Committee of the Catholic University of Pernambuco, under protocol # 092-2004/6-7. After the participants signed the informed consent form and the committee approved, we started to collect the data by means of a semi-directed interview, followed by the distribution of an information folder, which characterized the first stage of data collection.

The interview was made up of five questions, including the interviewee's knowledge regarding risk factors for hearing impairment, how knowledge was acquired, education regarding hearing health and risk factors, how this education was done. The risk factors described by the American Committee on Auditory Hearing Loss (Joint Committee on Infant Hearing) in 2008 are: family history of sensorineural hereditary hearing loss, maternal consanguinity, syndromes, congenital infections (rubella, syphilis, herpes, cytomegalovirus and toxoplasmosis) baby's craniofacial malformations, including the pinna, the external auditory canal, hyperbilirubinemia (with levels requiring exsanguinous transfusion), use of ototoxic medication such as: aminoglycosides, their association with diuretic agents and also chemotherapeutic agents, bacterial meningitis, Apgar scores between 0 and 4 on the 1st minute or 0 to 6 on the 5th minute, use of mechanical ventilation for more than five days, baby's ICU stay for more than forty-eight hours, recurrent or persistent otitis media for more than three months, suspicion of family members with speech and hearing delays and language delay, head trauma with loss of conscience or skull fracture.

On the following week, at the end of the interviews, we ran an educational campaign (educational talks given by one of the researchers), ending the first stage. The second stage, carried out with a minimum type of one month after the first stage, when the same interview was applied to the individuals participating on the first stage, in order to compare the studies.

For data and outcome analyses we used the 2003 Microsoft Office Excel, by means of a descriptive statistical analysis.

## RESULTS

Similar interviews were made of each health care professional in two steps: before and after the educational campaign. These interviews consisted of five questions; answers to each question were tabulated to show the frequency of responses to each alternative at both moments. Some subjects gave more than one answer to some of the questions.

Table 1 shows the results of professional knowledge about risk factors for hearing loss. In the first step, a higher citation frequency was seen for toxoplasmosis among gynecologists (73.84%) and rubella among pediatricians (92.29%). In the second moment, rubella was the most frequently cited factor by both gynecologists and pediatricians. A comparison of all factors at both moments showed that there was a general increase in the frequency of cited factors for both medical professionals. In the group of gynecologists, herpes, a low Apgar score, and cranial trauma were mentioned more often than other conditions; in the group of pediatricians, ototoxic medication; toxoplasmosis, cytomegalovirus, and bacterial meningitis became the most frequently mentioned factors. The item “others,” which comprises cited factors that are not risk factors for hearing loss in this study, went from 53.70% to 77.77%.

**Table 1:** Analysis of knowledge among health care professionals about the risk factors for hearing loss before and after a talk on this topic (educational campaign)

Risk factors	Before		After		Before		After	
	Gynecologists				Pediatricians			
	N	%	N	%	N	%	N	%
Family history of hearing loss	24	36,92%	42	64,61%	22	40,74%	39	72,22%
Maternal consanguinity	4	6,15%	11	16,92%	5	9,25%	20	37,03%
Rubella	43	66,15%	64	98,48%	50	92,59%	52	96,29%
Syphilis	27	41,53%	54	83,07%	16	29,62%	35	64,81%
Cytomegalovirus	32	49,23%	54	83,07%	26	48,14%	39	72,22%
Herpes	12	18,46%	35	53,84%	14	25,92%	22	40,74%
Toxoplasmosis	48	73,84%	62	95,38%	29	53,70%	41	75,92%
Craniofacial malformations	19	29,23%	41	63,07%	19	35,18%	31	57,40%
Hyperbilirubinemia	2	3,07%	12	18,46%	12	22,22%	25	46,29%
Ototoxic drugs	43	66,15%	51	78,46%	37	68,51%	48	88,88%
Bacterial meningitis	17	26,15%	33	50,76%	25	46,29%	34	62,96%
Apgar 0 to 4/1^st^ min or 0 to 6/5^th^ min	8	12,30%	31	47,69%	17	31,48%	36	66,66%
Mechanical ventilation > 5 days	3	4,61%	15	23,07%	6	11,11%	22	40,74%
ICU for over 48 hours	3	4,61%	24	36,92%	11	20,37%	30	55,55%
Recurring otitis media	13	20%	15	23,07%	18	33,33%	28	51,85%
Syndromes	2	3,07%	6	9,23%	6	11,11%	11	20,37%
Delayed language acquisition	4	6,15%	7	10,76%	4	7,40%	10	18,51%
Cranial trauma	18	27,69%	42	64,61%	13	24,07%	31	57,40%
Others	26	40%	26	40%	29	53,70%	42	77,77%
Total	65	100%	65	100%	54	100%	54	100%

Table 2 shows that the most frequent source of information was the undergraduate medical course for both medical specialists (80% of gynecologists and 75.92% of pediatricians).

**Table 2:** Knowledge acquisition mode of health care professionals

Answers	Before		After		Before		After	
	Gynecologists				Pediatricians			
	N	%	N	%	N	%	N	%
Talks	9	13,84	16	24,61	10	18,51	15	18,51
Courses	7	10,76	10	15,38	7	12,96	12	22,22
University	16	24,61	17	26,15	14	25,92	15	27,77
Medical training	56	86,15	49	75,38	41	75,92	44	81,48
Speech therapist	0	0	9	13,84	5	9,25	18	33,33
Others	4	6,15	37	56,92	7	12,96	30	55,55

Table 3 shows the number of health care professionals - in the first step - that routinely provided information about hearing health to their patients; only 20% of gynecologists did so, whereas 70.37% of pediatricians offered this information. These numbers increased in both groups at the second moment.

**Table 3:** Orientation given by health care professionals about auditory health.

Answers	Before		After		Before		After	
	Gynecologists				Pediatricians			
	N	%	N	%	N	%	N	%
Yes	35	53,84%	40	61,53%	38	70,37%	42	77,77%
No	30	46,15%	25	38,46%	16	29,62%	12	22,22%
Total	65	100,00%	65	100,00%	54	100,00%	54	100,00%

Table 4 shows information about orientation to families about risk factors for hearing loss; 41.54% of gynecologists and 61.12% of pediatricians answered yes to this item. Here, positive answers were more frequent at the second moment of the evaluation.

**Table 4:** Orientation of families about risk factors for hearing loss

Answers	Before		After		Before		After	
	Gynecologists				Pediatricians			
	N	%	N	%	N	%	N	%
Yes	26	40%	45	69,30%	33	61,11%	35	64,81%
No	39	60%	20	30,80%	21	38,88%	19	35,18%
Total	65	100%	65	100,00%	54	100,00%	54	100,00%

Table 5 shows how these professionals inform their patients; 46.15% of gynecologists and 53.70% of pediatricians used informal conversation as the method for conveying information. At the second moment of the study, the number of participants that used this procedure for conveying information was 67.69% for gynecologists and 66.66% for pediatricians.

**Table 5:** Type of orientation given by health care professionals

Answers	Before		After		Before		After	
	Gynecologists				Pediatricians			
	N	%	N	%	N	%	N	%
Talks	0	0,00%	1	1,53%	3	5,55%	3	5,55%
Conversations	26	40%	45	69,23%	29	53,70%	36	66,66%
Folders	0	0,00%	0	0,00%	0	0,00%	2	3,70%
Others	2	3,07%	1	1,53%	3	5.55%	3	5,55%

## DISCUSSION

The responses in this study were similar to those in other studies, as we will show next.

Congenital infection may be transmitted pre- and perinatally from mother to child, and may be avoided by providing information during prenatal visits. Lack of knowledge about hearing loss risk factors among gynecologists is a significant concern. There is still a high rate of deafness due to infection in our country; congenital infection is a major contributing factor in this context. These conditions are avoidable by dealing with environmental health conditions and providing medical care and training for health care professionals.

Table 1 shows the number of cited diseases that are not hearing loss risk factors; this is a picture of the available information about hearing health, and underlines the need for disseminating knowledge about hearing loss risks to foster prevention. A few authors have stated that the main reason explaining the incidence of deafness is lack of prevention of the main infections and contagious diseases.^20^^,^^9^ The relation between these factors - lack of knowledge and increased incidence of deafness - cannot be denied and points to the urgent need for intervening.^21^

The importance and efficacy of actions that promote hearing health has been demonstrated in a previous study;^22^ this is also evident in the present study as we look at the second step in the assessment, which showed the increased frequencies of all items.

Contrary to the arguments of Russo and Santos^5^ that health care professionals other than physicians first contact children postnatally, we argue that pediatricians should be the ones to identify children at risk for hearing problems as early as possible, since other health care professionals may not necessarily have the opportunity to contact with these children.

Although showing a higher rate of positive answers about health care orientation on hearing health and risk factors for hearing loss compared to gynecologists, pediatricians are not an absolute majority.^5^ This underlines the need for continued medical training and increasing their sensitivity to this topic. As we saw, the rates increased in the second moment of our study, showing that professionals probably understood the important of preventing the risk factors for deafness.

Neonatal hearing screening programs aim to diagnose hearing loss as early as possible in infancy, to undertake periodical monitoring for confirmation purposes, to identify progressive deafness and late manifestation of hearing loss, and to monitor the development of hearing.

For these goals to be met, parents should be adequately informed about the importance of caring for the hearing of their children.^23^

A high rate of evasion of parents is considered as the main hurdle against the success of hearing screening programs. Some reasons for parents not returning as recommended, are as follows: lack of information about the causes, symptoms and impact of hearing loss on the global development of their children; a common idea among mothers that their children are not at risk for hearing loss; and anxiety among mother when their children are being tested.^24^, ^25^, ^26^

The neonatal hearing screening program is part of the Early Detection of Child Hearing Loss project for users of the Unified Health System (SUS). Informing mothers about Neonatal Hearing Screening is part of the routine in visits; again, knowledge about this topic is paramount.^23^

Recent surveys have shown that professionals in the public health care system allege lack of resources, equipment and specific knowledge to investigate and monitor the development of hearing in children. Nevertheless, promotion measures are not costly but were not carried out routinely by the participants in our sample as shown at the first moment of this study.^25^^,^^27^

Speech therapists and otorhinolaryngologists have an important role in orienting medical professionals and counseling parents, as these specialists understand in detail the complexity of hearing screening and treatment of diagnosed cases.^21^

The purpose of providing knowledge about indicators of risk for hearing loss is to identify newborn that may require further care; this is of special relevance in developing countries where hearing screening is not available for every child. The aim is to reduce individual losses and public health costs.^25^^,^^28^^,^^29^

Health care problems are considered a social issue, not only because of their importance for quality of life, but because of the political and economic nature of health care within appropriate social and economic structures.^30^

It should be borne in mind that hearing health measures should include the following actions: promotion of health, surveillance, monitoring, and interventions. It is extensive work requiring a multidisciplinary approach.^31^, ^32^, ^33^

Initiatives to implement hearing screening programs remain a struggle for professionals dealing with hearing loss. Nevertheless, actions aiming at increasing collective awareness about this topic do not necessarily imply in new federal or state laws, and may be done rapidly and simply at a low cost. Interdisciplinarity and collective health measures should be fostered to reach such goals.^34^

Concurring with the present study, several authors have underlined the need for programs to disseminate information about neonatal hearing screening and the prevention of deafness; in this context, every professional whose work includes caring for the newborn may cooperate in the efforts to identify hearing loss at the ideal moment.^31^, ^32^, ^35^

## CONCLUSION

Our data revealed that generally the number of risk factors recalled by health care professionals was low, and that medical training has an important role in learning about this topic.

Orientation about risk factors for hearing loss and auditory health is given infrequently.

Promotional actions generate positive impact, which is evidenced by satisfactory increases in knowledge about this topic among the study sample. Thus, effective strategies need to be implemented so that risk factors for hearing loss may be identified, such as primary care for deafness programs, and the need for multidisciplinary work to extend information about this topic.
